# Successful Example of Implementing Screening of Liver Fibrosis in Specialist Diabetes Care

**DOI:** 10.1016/j.gastha.2024.10.017

**Published:** 2024-10-24

**Authors:** Muna Tajudin, Hannes Hagström, Sophia Rössner

**Affiliations:** 1 Division of Hepatology, Department of Upper GI, Karolinska University Hospital, Stockholm, Sweden; 2 Department of Medicine, Huddinge, Karolinska Institutet, Stockholm, Sweden; 3 Division of Endocrinology, Karolinska University Hospital, Stockholm, Sweden

**Keywords:** MASLD, Liver Fibrosis, Type 2 Diabetes Mellitus, Elastography

## Abstract

**Background and Aims:**

Patients with type 2 diabetes (T2D) constitute a risk group for presence and severity of metabolic dysfunction–associated steatotic liver disease (MASLD). Yet, there are few published examples of collaborations between endocrinologists and hepatologists in caring for patients with T2D and MASLD. Here, we describe a pathway for screening of liver fibrosis in routine specialist diabetes care at a tertiary care hospital.

**Methods:**

Patients with T2D seen at the Endocrinology department at Karolinska University Hospital, Stockholm, Sweden, during a structured intervention for T2D between October 2016 and September 2023 were eligible for inclusion. Liver stiffness measurements (LSM) and controlled attenuation parameter (CAP) as proxies for liver fibrosis and steatosis, respectively, were obtained utilizing vibration-controlled transient elastography (VCTE). An LSM cut-off to exclude advanced fibrosis was set to <8 kPa. Presence of MASLD was defined as a CAP value of CAP ≥ 294 dB/m.

**Results:**

A total of 177 patients with a valid LSM were included. The median age was 60 years and 60% were women. The median LSM was 5.8 (interquartile range 4.6–8.1) kPa, and the median CAP was 306 (258–362) dB/m. In total, 27% had LSM ≥8 kPa and 11% had LSM ≥12 kPa. MASLD was present in 55%. The clinical score for aspartate aminotransferase, alanine aminotransferase, age, platelet count had a low sensitivity for identifying patients with VCTE measurements above 8 kPa (34%) and 12 kPa (37%).

**Conclusion:**

This study provides an example of a productive partnership between endocrinologists and hepatologists using direct VCTE measurements, leading to the identification of a significant number of patients with presumed advanced fibrosis.

## Introduction

The prevalence of type 2 diabetes (T2D), overweight and obesity are increasing worldwide. It is estimated that globally, 529 million individuals lived with diabetes in 2021 and T2D accounted for 96% of these.^1^^,^^2^ Metabolic dysfunction-associated steatotic liver disease (MASLD) is a common comorbidity in T2D and is now the most common chronic liver disease affecting up to 38% of the global adult population.^3^

Metabolic dysfunction–associated steatohepatitis is the more severe form of MASLD and is associated with the development of liver fibrosis.^4^^,^^5^ Studies have shown the progression to fibrosis to be more common in patients with T2D compared to patients without.^6^^,^^7^ Approximately 65% of patients with T2D are estimated to have MASLD and 32% are estimated to have metabolic dysfunction–associated steatohepatitis.^8^

In turn, liver fibrosis is the main risk factor for the development of cirrhosis, hepatocellular carcinoma (HCC) and liver-related mortality.^9^, ^10^, ^11^, ^12^ The development of fibrosis is asymptomatic and often only apparent when progression to decompensated liver cirrhosis or HCC occurs. The gold standard for diagnosis of fibrosis is liver biopsy. However, this procedure is invasive, associated with risk of complication, subject to sampling error, time consuming and not cost-effective to be used as a screening tool.^13^ Several noninvasive tests (NITs) such as clinical score for aspartate aminotransferase (AST), alanine aminotransferase (ALT), age, platelet count (FIB-4), AST to platelets ratio index, and NAFLD fibrosis score have with varying sensitivity and specificity been proposed as instruments for screening of fibrosis.^14^ The accuracy of such NITs in secondary care has conflicting results, especially in patients with T2D.^15^

Vibration-controlled transient elastography (VCTE) is a simple, noninvasive method which uses ultrasound to calculate liver stiffness measurement (LSM) as a proxy for liver fibrosis and controlled attenuation parameter (CAP) as a proxy for liver steatosis.^14^^,^^16^ The LSM has been validated as reliable surrogate marker for liver fibrosis, and LSM values below 8.0 kPa have a high negative predictive value for excluding liver fibrosis.^14^^,^^17^

Following the increased risk of MASLD and fibrosis in patients with T2D, it might be beneficial to screen these patients to detect MASLD-related advanced fibrosis. The purpose of this is to allow lifestyle modifications and treatment to halt MASLD progression, thereby minimizing the risk of progression to decompensated liver cirrhosis. Patients with compensated cirrhosis may also be cared for by inclusion in screening programs for early detection of HCC, and to detect and treat portal hypertension, thereby reducing risk for primarily bleeding esophageal varices. Patients attending secondary diabetes clinics usually have more advanced T2D, and might constitute a subgroup where screening for liver fibrosis may be of particular value, since the prevalence of liver fibrosis among these patients has been estimated to 10%–20%, which is 2–4 times higher than in patients in primary care.^18^, ^19^, ^20^, ^21^ Patients with more advanced T2D also have a particularly high rate of progression to cirrhosis.^22^

Presently, there are some disparities among the guidelines concerning the use of NITs as screening tools. The current guidelines of the American Gastroenterology Association propose that patients with T2D besides a FIB-4 test, regardless of results, should also undergo a second assessment with either an imaging or biomarker-based NIT due to the high risk for hepatic fibrosis.^23^ The European Association for the Study of the Liver and the American Association for the Study of Liver Diseases as well suggest the computation of the FIB-4 score. However, the implementation of a secondary NIT, specifically VCTE, is recommended only when in patients with an elevated FIB-4 score, defined as above 1.3.^24^^,^^25^

Screening for other diabetes-related complications such as nephropathy and retinopathy is already routine in diabetes care. Since T2D can cause complications involving several organs, it is not uncommon for the patient to be followed up by for instance a cardiologist, nephrologist, ophthalmologist etc.^26^ Additional health care appointments might thus be demanding for the patient leading to decreased compliance. An optional approach is to include screening for organ complications in the existing infrastructure. Given the rising prevalence of MASLD, it becomes imperative to establish a robust collaboration between different specialists to efficiently detect and treat MASLD and associated complications.

Here, we describe how we implemented a pathway for screening of liver fibrosis in routine specialist diabetes care at the Endocrinology department in collaboration with Hepatology department of Karolinska University Hospital, Stockholm, Sweden, and the initial results.

## Method

### Participants

The care of patients with T2D in Stockholm County, Sweden is primarily provided at primary care centers. The Endocrinology Department of Karolinska University Hospital, Huddinge offers a 3-day long comprehensive assessment of cases considered complicated several times a year. During these days, around 8–15 patients participate in lectures by endocrinologists, dieticians, and diabetes nurses. Patients receive a personalized evaluation and treatment. The sources of referrals are mostly primary care centers, but other specialized clinics in Stockholm County also have the option of sending referrals.

Between October 2016 and September 2023, patients with diabetes type 2 aged ≥18 years that attended the Department of Endocrinology for an assessment were offered to also perform a VCTE at the Hepatology department. If the patient demonstrated values of 8 kPa or more during VCTE, an assessment by a hepatologist was offered.

Patients with known liver diseases such as viral hepatitis, hemochromatosis, primary biliary cholangitis, primary sclerosing cholangitis, autoimmune hepatitis, and drug-induced liver injury were excluded since those were already offered hepatology services. Excessive alcohol consumption (defined below) was also a cause for exclusion.

### Variables, Blood Workup, and Measurements

All patients underwent a clinical assessment by a doctor and nurse at the Endocrinology department. Pulse, blood pressure, weight and height were measured objectively. Body mass index was calculated as body weight (kilogram) divided by height (meter) squared. The occurrence of ongoing antidiabetic treatments and other medications was documented. Blood samples for analysis of total cholesterol, high-density and low-density lipoprotein, glycosylated hemoglobin (HbA1c), platelets and creatinine were taken after at least 8 hours of fasting. Previous or current smoking status was documented. Alcohol consumption was quantified by measuring phosphatidyl ethanol and calculating intake of alcohol unit by the Alcohol Use Disorders Identification Test or by history taking. Excessive alcohol consumption was defined as a score >8 points in the Alcohol Use Disorders Identification Test, phosphatidyl ethanol >0.3 μmol/L, or a reported alcohol intake >21 units for men and >14 units for women.

### Vibration-Controlled Transient Elastography

Liver stiffness measurement and controlled attenuation parameter was obtained utilizing VCTE (Fibroscan 502 Touch, EchoSens, Paris, France). The LSM values were considered reliable if the median was based on at least 10 successful acquisitions and with an interquartile range below 30% of the median if median values were above 7.1 kPa.^27^ The examinator started the measurements with the M-probe and if unsuccessful proceeded using the XL-probe.

Several studies have been conducted to finding the most accurate LSM cut-offs to rule out and rule-in significant liver fibrosis (stage 2–4). Most guidelines today suggest a cut-off for LSM <8 kPa to rule out advanced fibrosis, which yields a high sensitivity for significant fibrosis.^15^ Thus, this threshold of 8 kPa was applied here to exclude significant liver fibrosis. Furthermore, an upper cut-off at LSM ≥12 kPa was established in accordance with the suggestions by guidelines regarding advanced fibrosis.^24^ The optimal CAP value as cut-off to define the presence of hepatic steatosis has been studied to a lesser extent. Here, we determined the cut-off at CAP ≥ 294 dB/m to distinguish between steatosis grade 0 to steatosis grade 1–3 based on the publication by Petroff et al and in accordance with the manufacturer’s suggested cut-off value.^28^

### Statistical Analysis

Continuous variables are presented as medians with interquartile ranges and categorical variables are presented as total numbers and percentages. The difference between continuous and categorical variables was assessed respectively using Wilcoxon rank sum test and Fisher’s exact test. The negative predictive value and positive predictive value as well as the sensitivity and specificity of the FIB-4 score to predict elevated LSM values at the cut-off of ≥8 kPa and ≥12 kPa were calculated. A FIB-4 score of ≥1.3 for individuals in the age range 36–64 years and FIB-4 score >2.0 for individuals ≥65 years was deemed elevated.^29^ A *P* value <.05 was considered significant. All statistical analyses were conducted using Stata, version 17.0 (Stata Corp LLC, College Station, TX).

## Results

### Practical Considerations for Pathway Development

**Figure 1:**
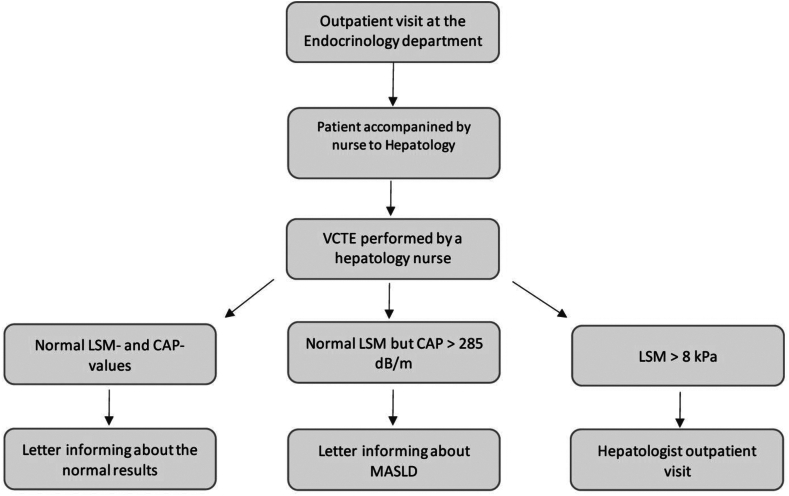
Flowchart of the screening pathway.

The Endocrinology department agreed to routinely obtain AST and ALT measurements in their care package for T2D, allowing for FIB-4 calculations. The initial practical approach we used was to transport the Fibroscan device to the Endocrinology department and perform measurements on site. This was well received by patients but led to hardware malfunction due to the transportation. As a next step, we instead instructed nurses at the Endocrinology department to guide patients with T2D to the Hepatology department outpatient clinic, around 300 m (Figure 1). Here, a hepatology nurse performed the VCTE examination. Patients with a CAP value < 285 dB/m, and a liver stiffness <8 kPa were given a short letter informing them about the benign nature of the exam. Patients with a CAP value of ≥285 dB/m but a liver stiffness <8 kPa were given a short letter informing them about the presence of MASLD but reassuring them they had a very low risk to have liver fibrosis. Finally, patients with a liver stiffness ≥8 kPa were given a short letter informing them about an elevated liver stiffness and the need for additional examinations. These patients were then offered a hepatologist outpatient visit.

For integrity and privacy reasons, patients that did not want to undergo VCTE could not be included to compare baseline characteristics and are therefore not included in the analysis.

### Results of Pathway in Case Finding

**Figure 2:**
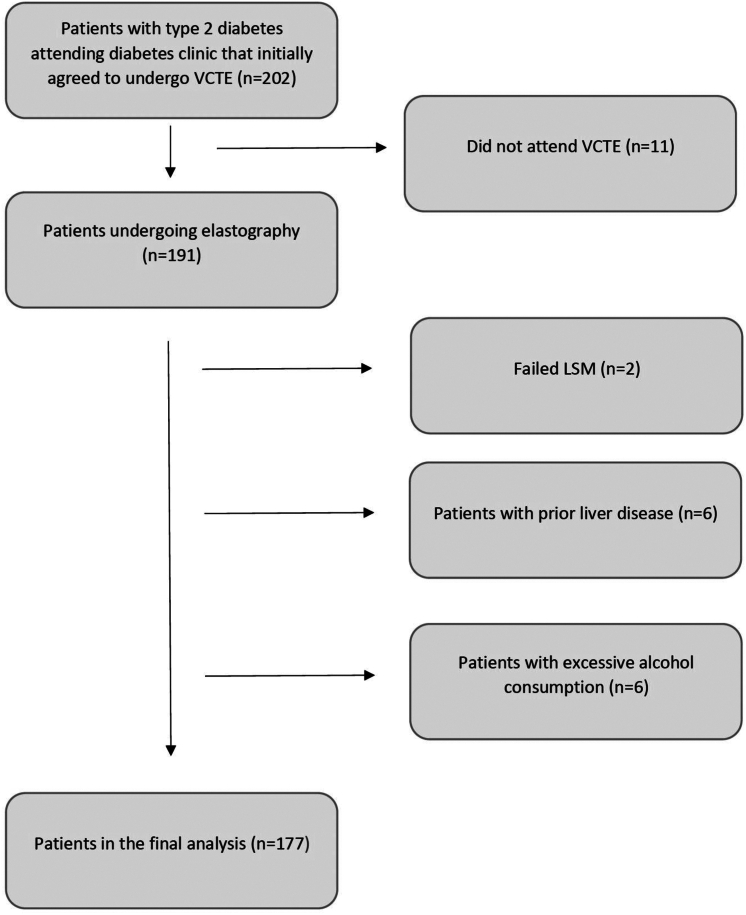
Flowchart of the inclusion and exclusion of patients.

A total of 177 patients with a valid LSM were included (Figure 2). The median age was 60 years and 60% were women. The median LSM was 5.8 (95% confidence interval (CI) = 4.6–8.1) kPa, and the median CAP was 306 (95% CI = 258–362) dB/m (Table 1). In total, 27% had LSM ≥8 kPa and 11% had LSM ≥12 kPa. Higher values of CAP, body mass index, ALT and FIB-4 score and lower levels of high-density lipoprotein were seen in the group with LSM ≥8 kPa compared to the group with LSM <8 kPa. Similar differences were present when comparing the group with LSM ≥12 to the group with LSM <12 kPa. A difference between the groups with a smaller proportion ongoing metformin treatment and larger probe size were only present with a cut-off at 12 kPa (Table 2). Baseline characteristics stratified on liver stiffness cut-offs of 8 and 12 kPa respectively, are presented in Table 2. Baseline characteristics stratified on CAP cut-off of 294 dB/m are presented in Table 3.

**Table 1:** Baseline Characteristics of the Included Patients, Data Presented as Medians With Interquartile Range, or Total Numbers With Percentages

Characteristics	Total	Missing (%)
	N = 177	
Age (y)	60 (50–68)	0
Sex, female n (%)	106 (59.9%)	0
CAP (dB/m)	306 (258–362)	12
LSM (kPa)	5.8 (4.6–8.1)	0
BMI	29.9 (26.7–34.3)	2
HbA1c (mmol/mol)	68 (58–80)	2
Hypertension, n (%)	121 (68.4%)	0
Hyperlipidemia, n (%)	102 (57.6%)	0
Insulin treatment, n (%)	113 (63.8%)	0
Metformin treatment, n (%)	114 (64.4%)	0
Systolic blood pressure (mmHg)	135 (125–148)	4
Probe size large, n (%)	98 (55.4%)	3
Creatinine (μmol/L)	73 (60–93)	2
Fasting glucose (mg/dl)	9.3 (7.6–11.5)	36
Platelet count (10ˆ9/L)	250 (207–308)	3
Triglyceride (mmol/L)	1.80 (1.10–2.60)	6
High-density lipoprotein (mmol/L)	1.00 (0.80–1.30)	7
Low-density lipoprotein (mmol/L)	2.34 (1.60–3.12)	18
AST (U/L)	0.40 (0.33–0.52)	27
ALT(U/L)	0.49 (0.38–0.71)	23
FIB-4 score	1.01 (0.71–1.37)	24
Smoking		0
Nonsmoker, n (%)	84 (47.5%)	
Former smoker, n (%)	78 (44.1%)	
Current smoker, n (%)	15 (8.5%)	

BMI, body mass index.

**Table 2:** Baseline Characteristics Stratified on a LSM Measurement of 8 kPa and 12 kPa

Characteristics	Total	LSM < 8 kPa	LSM ≥ 8 kPa	*P* value	LSM < 12 kPa	LSM ≥ 12 kPa	*P* value 2
	N = 177	N = 129	N = 48		N = 157	N = 20	
Age (y)	60 (50–68)	59 (50–68)	60 (50–67)	.77	60 (50–67)	63 (48–70)	.56
Sex, female n (%)	106 (59.9%)	77 (59.7%)	29 (60.4%)	.93	62 (39.5%)	9 (45.0%)	.64
CAP (dB/m)	305 (258–362)	292 (251–347)	339 (291–384)	**.004**	297 (253–352)	378 (317–400)	**<.001**
BMI	29.9 (26.7–34.3)	29.1 (25.8–32.5)	33.0 (28.9–36.9)	**<.001**	29.4 (26.5–33.1)	34.6 (30.4–40.4)	**<.001**
HbA1c (mmol/mol)	68 (58–80)	68 (58–81)	70 (60–79)	.8	68 (58–80)	73 (60–79)	.75
Hypertension, n (%)	121 (68.4%)	83 (64.3%)	38 (79.2%)	.059	106 (67.5%)	15 (75.0%)	.5
Hyperlipidemia, n (%)	102 (57.6%)	71 (55.0%)	31 (64.6%)	.25	87 (55.4%)	15 (75.0%)	.095
Insulin treatment, n (%)	113 (63.8%)	84 (65.1%)	29 (60.4%)	.56	102 (65.0%)	11 (55.0%)	.38
Metformin treatment, n (%)	114 (64.4%)	86 (66.7%)	28 (58.3%)	.3	106 (67.5%)	8 (40.0%)	**.015**
Systolic blood pressure (mmHg)	136 (125–148)	135 (125–150)	138 (125–144)	.86	135 (125–150)	139 (127–146)	.69
Probe size large, n (%)	98 (55.4%)	68 (52.7%)	30 (62.5%)	.34	84 (53.5%)	14 (70.0%)	**.023**
Creatinine (μmol/L)	73 (60–93)	72 (61–93)	75 (55–95)	.93	73 (61–93)	77 (52–99)	.95
Fasting glucose (mg/dl)	9.3 (7.6–11.5)	9.2 (7.1–11.9)	9.8 (8.0–11.5)	.28	9.4 (7.4–11.6)	9.2 (8.2–11.5)	.7
Platelet count (10ˆ9/L)	250 (207–308)	248 (207–297)	251 (199–312)	.97	251 (207–308)	248 (201–296)	.79
Triglyceride (mmol/L)	1.80 (1.10–2.60)	1.70 (1.10–2.65)	2.00 (1.60–2.50)	.11	1.70 (1.10–2.60)	2.05 (1.65–2.65)	.28
High-density lipoprotein (mmol/L)	1.0 (0.8–1.3)	1.1 (0.9–1.4)	0.9 (0.8–1.1)	**.008**	1.1 (0.9–1.4)	0.9 (0.8–1.0)	**.011**
Low-density lipoprotein (mmol/L)	2.3 (1.6–3.1)	2.4 (1.8–3.2)	2.3 (1.4–2.9)	.22	2.4 (1.8–3.2)	1.9 (1.4–3.1)	.21
ALT(U/L)	0.49 (0.38–0.71)	0.46 (0.34–0.66)	0.59 (0.42–0.87)	**.011**	0.48 (0.35–0.70)	0.63 (0.47–0.82)	**.025**
FIB-4 score	1.01 (0.71–1.37)	0.93 (0.68–1.28)	1.19 (0.86–1.71)	**.007**	0.95 (0.69–1.28)	1.22 (0.86–1.82)	**.035**
Smoking							.5
Nonsmoker, n (%)	84 (47.5%)	61 (47.3%)	23 (47.9%)	.83	76 (48.4%)	8 (40.0%)	
Former smoker, n (%)	78 (44.1%)	58 (45.0%)	20 (41.7%)		69 (43.9%)	9 (45.0%)	
Current smoker, n (%)	15 (8.5%)	10 (7.8%)	5 (10.4%)		12 (7.6%)	3 (15.0%)	

Data are presented as medians with interquartile range, or total numbers with percentages.

Bold values signify <.05.

**Table 3:** Baseline Characteristics Stratified on CAP Measurement of 294 dB/M

Characteristics	Total	CAP <294 dB/m	CAP ≥ 294 dB/m	*P* value
	N = 156	N = 70	N = 86	
Age (y)	60 (50–69)	61 (53–71)	59 (49–65)	.08
Sex, female n (%)	63 (40.4%)	26 (37.1%)	37 (43.0%)	.46
CAP (dB/m)	306 (258–362)	252 (222–274)	355 (333–384)	**<.001**
LSM (kPa)	5.8 (4.4–8.1)	5.2 (4.0–6.4)	6.9 (5.3–8.9)	**<.001**
BMI	29.6 (26.4–34.2)	27.3 (24.8–29.2)	32.6 (29.4–35.9)	**<.001**
HbA1c (mmol/mol)	68 (59–80)	64 (55–75)	71 (61–82)	**.025**
Hypertension, n (%)	111 (71.2%)	47 (67.1%)	64 (74.4%)	.32
Hyperlipidemia, n (%)	93 (59.6%)	44 (62.9%)	49 (57.0%)	.46
Insulin treatment, n (%)	101 (64.7%)	51 (72.9%)	50 (58.1%)	.056
Metformin treatment, n (%)	101 (64.7%)	35 (50.0%)	66 (76.7%)	**<.001**
Systolic blood pressure (mmHg)	138 (125–150)	133 (125–150)	140 (125–150)	.42
Probe size large, n (%)	83 (53.2%)	24 (34.3%)	59 (68.6%)	**<.001**
Creatinine (μmol/L)	73 (61–94)	73 (63–98)	72 (58–93)	.45
Fasting glucose (mg/dl)	9.2 (7.6–12.6)	8.4 (6.4–10.8)	10.1 (8.2–12.6)	.026
Platelet count (10ˆ9/L)	250 (207–297)	243 (207–315)	253 (206–296)	.96
Triglyceride (mmol/L)	1.80 (1.20–2.80)	1.35 (0.95–1.90)	2.30 (1.60–3.30)	**<.001**
High-density lipoprotein (mmol/L)	1.0 (0.8–1.3)	1.1 (0.9–1.4)	1.0 (0.8–1.2)	.062
Low-density lipoprotein (mmol/L)	2.3 (1.6–3.1)	2.5 (1.6–3.2)	2.3 (1.6–3.1)	.78
ALT (U/L)	0.48 (0.35–0.71)	0.40 (0.32–0.54)	0.58 (0.41–0.83)	**<.001**
FIB-4 score	0.96 (0.71–1.31)	0.94 (0.57–1.50)	1.04 (0.75–1.24)	.41
Smoking				.12
Nonsmoker, n (%)	79 (50.6%)	41 (58.6%)	38 (44.2%)	
Former smoker, n (%)	63 (40.4%)	22 (31.4%)	41 (47.7%)	
Current smoker, n (%)	14 (9.0%)	7 (10.0%)	7 (8.1%)	

Bold values signify <.05.

BMI, body mass index.

MASLD was present in 86 (55%) patients. Patients with MASLD had a median LSM of 6.9 (5.3–8.9) kPa compared to 5.2 (4.0–6.4) kPa for individuals with CAP <294 dB/m (*P* < .001). Patients with MASLD had higher values of ALT, HbA1c, and triglycerides than patients without MASLD (Table 3).

Out of 177 patients, 76% had all necessary parameters available to calculate a FIB-4 score, with missing values being due to initial miscommunication of lab order packages. Within the patients with an LSM above 8 kPa, 14 also had elevated FIB-4 score which generated a sensitivity of 34% (95% CI = 20–50). Test characteristics using the 8 kPa cut-off are presented in Table 4.

**Table 4:** 2 × 2 Table of FIB-4 and Liver Stiffness Measurement With a Cut-Off at 8 kPa

Measurement	Elevated FIB-4 score	Normal FIB-4 score	
≥8 kPa	14	27	Sensitivity 34% (20–50)
<8 kPa	9	86	Specificity 91% (83–96)
	PPV 61% (39–80)	NPV 76% (67–84)	

Elevated FIB-4, score defined as ≥1.3 for individuals in the age range 36–64 y and FIB-4, score >2.0 for individuals ≥65 y. Sensitivity, specificity, positive predictive value, and negative predictive value for FIB-4, score to detect elevated liver stiffness of 8 kPa or more.

NPV, negative predictive value; PPV, positive predictive value.

A corresponding analysis was performed using a cut-off value of 12 kPa. Of those with LSM ≥12 kPa, 7 individuals also had an elevated FIB-4 score resulting in a sensitivity of 37% (95% CI = 16–62). Test characteristics using the 12 kPa cut-off are presented in Table 5.

**Table 5:** 2 × 2 Table of FIB-4 and Liver Stiffness Measurement With a Cut-Off at 12 kPa

Measurement	Elevated FIB-4 score	Normal FIB-4 score	
≥12 kPa	7	12	Sensitivity 37% (16–62)
<12 kPa	16	101	Specificity 86% (79–92)
	PPV 30% (13–53)	NPV 89% (82–94)	

Elevated FIB-4, score defined as ≥1.3 for individuals in the age range 36–64 y and FIB-4, score >2.0 for individuals ≥65 y. Sensitivity, specificity, positive predictive value, and negative predictive value for FIB-4, score to detect elevated liver stiffness of 12 kPa or more.

NPV, negative predictive value; PPV, positive predictive value.

Forty-eight patients with LSM of more than 8 kPa were offered an outpatient consultation with a hepatologist. Among these, 3 patients declined follow-up, and 4 were lost to follow-up. Of the remaining patients who established care, 6 were confirmed to have cirrhosis based on a repeated liver stiffness measurement of more than 12 kPa and overall hepatologist evaluation.

A further total of 10 patients underwent liver biopsy, with fibrosis classified according to the Metavir scoring system: 1 patient had Metavir F3, 3 had Metavir F2, and 5 had Metavir F1. One patient opted against biopsy and instead underwent magnetic resonance elastography, which indicated F2 fibrosis. Notably, 1 patient progressed to decompensated liver cirrhosis.

## Discussion

This study details the integration of liver fibrosis screening into routine diabetes patient care within a university hospital setting. Our findings reveal a substantial proportion of queried patients who willingly participated in the examination. Additionally, we observe a sizable prevalence of MASLD in this population at 55%. This figure is somewhat lower than the reported 65% prevalence in T2D from a recent meta-analysis.^30^ It is notable that this figure was despite that we investigated patients with a more severe disease, as evidenced by high HbA1c levels and frequent comorbidities. Concerning the prevalence of liver fibrosis, 11% had advanced fibrosis, defined by a VCTE exam of 12 kPa or higher. This indicates that it may be reasonable to screen such “advanced” populations using VCTE directly. Further motivation of this is that we investigated the FIB-4 score to identify patients with advanced fibrosis, finding a low sensitivity of around 37%. This suggests that reliance on FIB-4 may not be advisable in specialized diabetes care, and direct VCTE might be a more suitable approach.

A study worth mentioning for discussion include a group of 330 patients with similar characteristics (T2D, followed at a tertiary clinic) studied by Gautier et al^31^ These patients underwent an alternative approach to investigation through biopsy if they had consistently elevated levels of ALT—above 30 IU/L for men and above 20 IU/L for women. This approach involved a low threshold for biopsy, but some patients had already undergone radiological examinations that confirmed the presence of steatosis within this group. The prevalence of advanced fibrosis (F3-F4) was found to be 38%, and nonalcoholic steatohepatitis was observed in 58% based on the biopsies.

The median ALT level in our study was 49 IU/L, indicating that at least half of the patients would be eligible for biopsy, although the value was missing in 23% of the patients. Notably, the distribution of ALT values between patients with LSM greater than 8 kPa and those with LSM greater than 12 kPa consistently exceeded 40 IU/L for all groups. Therefore, at least half of the patients with LSM < 8 kPa would be eligible for biopsies based on this criterion.

As noted by Gautier et al., the prevalence of advanced fibrosis may be underestimated in studies utilizing VCTE, as ours, compared to if patients had also undergone biopsies using the same criteria. Alternatively, the prevalence would remain essentially unchanged due to the stated differences in patient selection. Nevertheless, the biopsy verified findings of a high prevalence of advanced fibrosis underscore the need to screen patients with T2D at the tertiary level.

Our study’s strength include a moderately large sample size of patients with advanced T2D from a university hospital setting and the establishment of a collaboration lasting a considerable 6 years, briefly interrupted by the COVID-19 pandemic, illustrating its feasibility. However, initial miscommunication led to missing data on ALT and AST for 23% and 27%, highlighting the need for clear communication between involved parties. Since the start of the study, a new classification of steatotic liver disease has been established. Metabolic dysfunction–associated steatotic liver disease with moderate alcohol intake has emerged as a new entity which includes patients with MASLD *and* a weekly alcohol consumption of 140–350 g for females or 210–420 g for males. We excluded patients with an alcohol intake corresponding to approximately 210 g for males and 140 g for females but lack further information of consumption in the remaining patients which could mean in a selected few that they might have had metabolic dysfunction–associated steatotic liver disease with moderate alcohol intake. Finally, due to legal and administrative reasons, not all patients attending the diabetes clinic could be included in the analysis of eligible patients in the study flowchart, and there is some risk of selection bias.

Many hospitals in Sweden already have in place a comprehensive week-long assessment of patients with diabetes, akin to the approach at Karolinska University Hospital. Commonly integrated in the hospital setting is also a gastroenterology and hepatology clinic, usually with the access to VCTE and hepatological competence. Our findings suggest that adopting a similar setup may be both possible to introduce at such hospitals, in Sweden and similar countries. The potential of identifying a substantial proportion of patients with probable cirrhosis to include in the surveillance of HCC is supported by our results.

Recently, the American Diabetes Association published new recommendations of patients with MASLD and T2D to be prioritized for the treatment with glucagon-like peptide-1 analogs based on findings in a phase II study.^32^ Given the recent approval of Resmetirom to treat MASLD,^33^ it is essential to identify eligible patients to start treatment timely.

## Conclusion

In summary, this cross-sectional study suggests that it is possible to develop a fruitful collaboration between endocrinologists and hepatologists at a major university hospital, with the pathway leading to identification of a considerable proportion of patients with MASLD and with a high prevalence of elevated liver stiffness suggesting advanced fibrosis.
